# Micro-endoscopic *In Vivo* Monitoring in the Blood and Lymphatic Vessels of the Oral Cavity after Radiation Therapy

**DOI:** 10.7150/ijms.36470

**Published:** 2019-10-21

**Authors:** Mi Ran Byun, Seok Won Lee, Bjorn Paulson, Sanghwa Lee, Wan Lee, Kang Kyoo Lee, Yi Rang Kim, Jun Ki Kim, Jin Woo Choi

**Affiliations:** 1 Department of Pharmacology, College of Pharmacy, Kyung Hee University, Seoul, 02447, Republic of Korea; 2 Department of Life and Nanopharmaceutical Science, Graduate School, Kyung Hee University, Seoul, 02447, Republic of Korea; 3 Biomedical Engineering Research Center, Asan Institute for Life Sciences, Asan Medical Center, University of Ulsan College of Medicine, Seoul, 05505, Republic of Korea; 4 Department of Oral and Maxillofacial Radiology, College of Dentistry, Wonkwang University, Iksan, 54538, Republic of Korea; 5 Department of Radiation Oncology, School of Medicine, Wonkwang University, Iksan, 54538, Republic of Korea; 6 Department of Hemato-Oncology, Yuseong Sun Hospital, Daejeon, 34084, Republic of Korea; 7 Department of Convergence Medicine, University of Ulsan College of Medicine, Seoul, 05505, Republic of Korea

**Keywords:** head and neck cancer, radiotherapy, mouse models, microendoscopy, fluorescence imaging

## Abstract

Radiotherapy, although used worldwide for the treatment of head, neck, and oral cancers, causes acute complications, including effects on vasculature and immune response due to cellular stress. Thus, the ability to diagnose side-effects and monitor vascular response in real-time during radiotherapy would be highly beneficial for clinical and research applications. In this study, recently-developed fluorescence micro-endoscopic technology provides non-invasive, high-resolution, real-time imaging at the cellular level. Moreover, with the application of high-resolution imaging technologies and micro-endoscopy, which enable improved monitoring of adverse effects in GFP-expressing mouse models, changes in the oral vasculature and lymphatic vessels are quantified in real time for 10 days following a mild localized single fractionation, 10 Gy radiotherapy treatments. Fluorescence micro-endoscopy enables quantification of the cardiovascular recovery and immune response, which shows short-term reduction in mean blood flow velocity, in lymph flow, and in transient immune infiltration even after this mild radiation dose, in addition to long-term reduction in blood vessel capacity. The data provided may serve as a reference for the expected cellular-level physiological, cardiovascular, and immune changes in animal disease models after radiotherapy.

## Introduction

In patients with cancers of the head and neck, radiotherapy is needed not only to prevent the recurrence of residual tumours after surgical treatment, but also to treat patients presenting with operable tumours or multiple lesions. However, radiotherapy of the head and neck commonly causes significant adverse reactions. Although the severity of side effects varies based on the total radiation dose and fractionation schedule, numerous complications are commonly observed upon irradiation 1-4. In human patients, these side effects have been grouped into three categories: acute symptoms, such as drowsiness, headache, and emesis; early-delayed symptoms, such as mucositis, nausea and diarrhoea; and late effects, which include pulmonary fibrosis, atrophy, vascular and neural damage, and the development of secondary malignancies 3.

The symptoms of head and neck radiotherapy may also be grouped based on their severity: in addition to mild complications of the hair and skin 5,6, mild complications of the oral cavity have also been observed, including oral mucositis, dermatitis, and parotitis with tissue damage 6-8. These complications may be related to inflammation in the oral cavity. More serious toxicity to central nervous system (CNS) tissues and cerebrovascular diseases, such as intracranial neoplasm, tumors 1,4, occlusive vascular disease and stroke, intracranial haemorrhage, cavernous malformations, and changes in the vasculature 9 are some of the possible irreversible complications. Therefore, for vascular diseases the diagnosis, prevention, and treatment of these diseases are more important than complication management.

Mouse models are commonly used for study of the side effects of radiotherapy, due to ease of handling and accelerated experimental timeframes. While the development of oral mucositis takes up to six weeks in human patients being treated for head and neck cancers 10, it appears in four days after a single fractionation dose in mice 11. Similarly, long-term effects such as pulmonary fibrosis develop over 12 weeks after a 20 Gy treatment in mice 12, and vasculature changes such as increased permeability of the blood-brain barrier develop over 90 days in a 40 Gy fractionated murine model 13. While treatment of human patients is generally fractionated over several weeks, experiments on mouse tumour models are commonly completed in between 1 and 3 fractions, and vascular recovery is observed after 11 to 13 days 14. A single fractionation of 5-10 Gy has been observed to be appropriate for the observation of mild vascular damage in murine models 14.

Recently, new imaging methods that can visualize the early change in vasculature after radiotherapy have resulted from advances in fibre optic and micro-optical instrumentation 15,16. The miniaturizing of optical systems and high-resolution imaging technologies could help in minimally invasive procedures and provide high-quality intra-vital images. For example, miniaturized fluorescence endoscopy has been used pre-clinically to provide an enhanced and detailed image of the mucosa surface 17. Moreover, it may be used to classify the vasculature around tumorous lesions during tumorigenesis 17-20. When these advanced techniques are used, physiological changes in the vascular or lymphatic systems can be visualized at high resolution through non-invasive methods.

In this study, changes in the blood vessels and lymphatic system have been intra-vitally monitored using fluorescence endoscopic techniques in the oral cavity following the use of single fractionation radiotherapy to cause a mild vascular response and recovery, without the application of any other physical trauma. Transgenic mice expressing green fluorescent protein (GFP) in their blood and lymph vasculature allow for the non-invasive quantification of the tissue response to radiotherapy at the cellular level. Measurement of the changes in vasculature fluorescence, immune fluorescence, and blood flow velocity following the injection of fluorescent dyes, has resulted in a novel view into the *in vivo* immune response and recovery of vascularization following radiotherapy. In addition to short-term reduction in mean blood flow, in lymph flow, and a transient immune response, long-term reduction in blood vessel capacity is observed through fluorescence, even after this mild radiation dose.

## Materials and Methods

### Experimental design and suction setup

**Figure 1:**
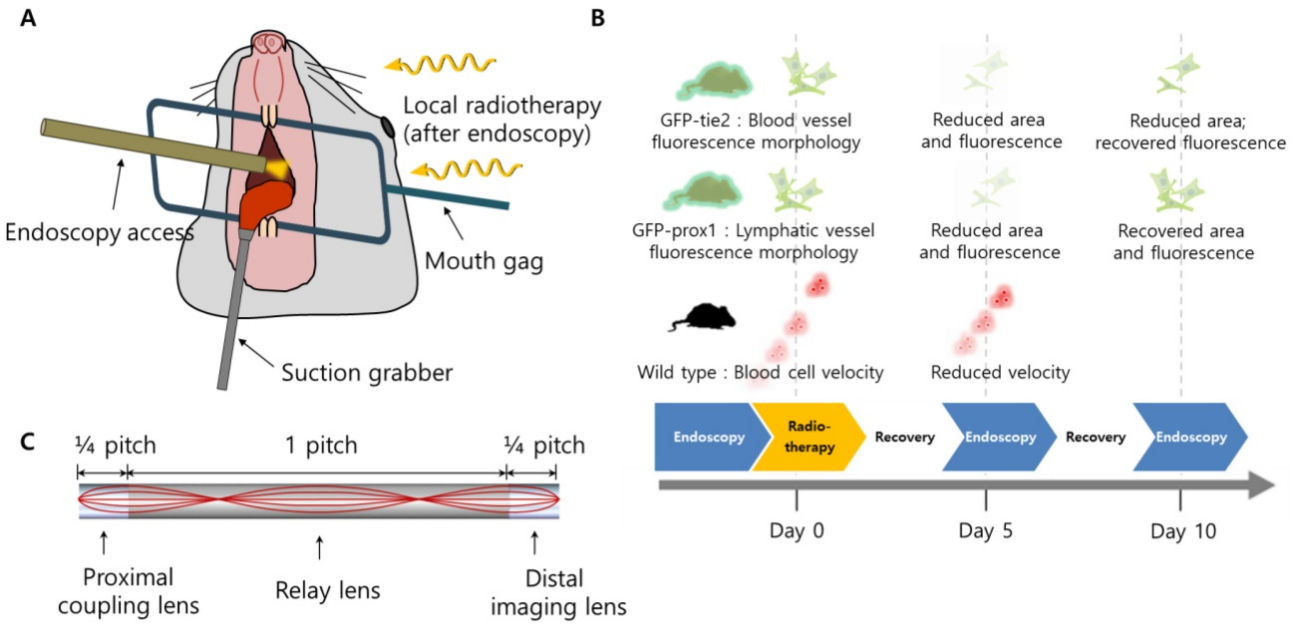
Schematics of the study. (A) The setup for oral radiotherapy and micro-endoscopic intravital imaging of the mouse buccal mucosa. (B) Radiotherapy schedule and a summary of significant observation. (C) Design of triplet GRIN endoscope.

A schematic illustration of the setup for oral radiotherapy and intra-vital cheek monitoring is shown in Figure 1(a), while the timeline of experiments is shown in Figure 1(b). A customized stainless steel mouth gag was placed between the upper and lower teeth of the anesthetized mouse to keep its mouth open, after which a small suction tube with an inner diameter of 2.0 mm was used to secure the tongue out of the mouth of the anesthetized mouse for radiation therapy and clear micro-endoscopic imaging. Suction pressure of about 25 mmHg was used to hold the mouse tongue securely without causing tissue damage. With the oral cavity opened and tongue immobilized, micro-endoscopic imaging and radiation therapy were performed sequentially following the experimental schedule of Figure 1(b). Artificial saliva was sprayed on the tongue and cheek in 5 minute intervals to maintain the physiological aqueous environment during imaging.

### Mouse models

Fifteen female mice, aged 6 to 10 weeks old, and expressing *GFP-tie2* (Jackson Laboratory), *GFP-prox1* (Jackson Laboratory), or wild type, were used 21,22, with five mice of each variant in each of the control and treatment groups. The mice were anesthetized intraperitoneally with ketamine (90 mg/kg) and xylazine (9 mg/kg), which were mixed with body-temperature phosphate buffered saline before injection.

### Mouse radiotherapy procedure

Irradiation was applied to mice under general anesthesia with ketamine and xylazine, as described above, to the head area as a single dose, 0 Gy (control group, n = 15), 10 Gy (treatment group, n = 15), using a linear accelerator (Clinac iX, Version 7.5. Varian Medical Systems, USA) with a 6-MV X-ray beam at a dose-rate of 2 Gy/min. This dosage is sufficient to induce some symptoms of radiotherapy, but weak enough to avoid mucositis, which may have an adverse effect on imaging. To shield the lung and abdomen of the mice, the radiation field was attenuated with a lead block. For delivery of maximal radiation doses to the mice, the head of the mice were covered with a bolus 1.5 cm thick, and the mice were placed on an acryl phantom more than 15 cm thick. In order to properly shield and model human radiation dosage, radiation was delivered from the top of the mouse head downward.

### *In vivo* endoscopic imaging of the blood and monitoring of the lymphatic vessels

The mice were also anesthetized with ketamine and xylazine for *in vivo* imaging sessions, following the same procedure as for radiation described above. In order to avoid suffocation and aid in the capture of clear images, the tongue was gently pulled out from the oral cavity using a miniature mouth gag and tongue suction system (Figure 1). Mice were imaged in the fluorescent modality, using mice expressing *GFP-tie2* and *GFP-prox1* for the imaging of blood and lymphatic vessels, respectively.

A micro-endoscope of diameter 1.0 mm was used to observe changes in the blood vessels and lymphatic vessels in the buccal mucosa of the oral cavity. The micro-endoscope was fabricated for minimally invasive imaging using a gradient index (GRIN) lens triplet to a final diameter of 1.0 mm and a length of 5 cm, a field of view of 195 µm, and was combined by means of an attachable relay to a home-built confocal micro-endoscope system 17,23. The home-built laser scanning confocal system consists of two galvano-scanner mirrors that sweep over each frame of 512 by 512 pixels at 30 Hz for real-time intra-vital imaging. The system was excited by a 488 nm laser source for visualization of GFP fluorescence in the blood and lymphatic vessels of the transgenic *GFP-tie2+* and *GFP-prox1+* mice. A 532 nm laser source was used to excite rhodamine-B dextran in wild type mice for blood flow analysis. The confocal setup had two different detection channels, consisting of photomultiplier tubes (PMT) filtered to detection ranges of 525 nm ± 25 nm and 607 nm ± 18 nm, corresponding to the emission ranges for GFP and rhodamine-B, respectively. For all endoscopy experiments, the light sources were maintained at the same power, and the PMT conditions were calibrated to maintain the same sensitivity despite measurements being separated by several days. Tissue auto-fluorescence was eliminated in wild-type mice by adopting narrow-bandwidth optical filters in front of the PMT detectors.

### Histological evaluation

Histological evaluations were performed to confirm immune cell infiltration into the tissues after radiotherapy. After euthanasia, excised tissue from the mouse buccal mucosa was fixed with 10% formalin for 48 hours or longer and embedded in paraffin, before section slices were prepared. Paraffin-sectioned slices were stained with CD4+, CD8+, and F4/80 antibodies for immune cell imaging. Samples were visualized through conventional fluorescence microscopy (Olympus, Japan).

### Vascular flow analysis

Mean blood flow velocity was measured by the analysis of video footage from micro-endoscopic measurements of the wild-type mouse cheek. For the measurement of mean blood flow velocity, rhodamine-B dextran (70 kDa, Sigma) was injected and used to visualize the blood vessels. In post-processing, erythrocytes were identified and tracked manually over the course of 0.2 s of video. For each wild-type mouse, red blood cells were tracked from different positions, resulting in a total of 5 measurements during each measurement day. Vascular flow data was presented as mean distance ± standard error in the mean over 5 measurements.

### Animal experiments

All animal experiments were performed according to protocols approved by the Institutional Animal Care and Use Committee (IACUC) of the Wonkwang University. The committee followed the guidelines set by the New York Academy of Sciences Ad Hoc Animal Research Committee and by the Institute of Laboratory Animal Resources (ILAR).

### Cell preparation

Primary lung fibroblast WI-38 and gingival fibroblast HGF were kindly provided by the laboratory of Prof. S. Park of Wonkwang University (Jeonbuk, South Korea). Primary endothelial cells HUVEC and HCAEC were purchased from American Type Culture Collection (ATCC; VA, US). The cells were cultured in complete endothelial cell growth medium with heparin solution (Sigma H3393), using endothelial cell growth supplement (BD Bioscience 354006) for HUVEC and MEM for WI38 in RPMI 1640 (Hyclone, US) with 10% fetal bovine serum (Hyclone, US), 200 μg/ml penicillin and 100 μg/ml streptomycin at 37°C and 5% CO_2_.

### Culture radiation procedure

Cultured cells in complete medium were sealed in plate for irradiation by either 0 Gy or 10 Gy of gamma rays generated by a caesium-137 irradiator.

### Real-time PCR

RNA was purified from each of the cell samples before and after irradiation using ethanol precipitation. To analyse the expression level of human *Tie2* (TEK receptor tyrosine kinase) mRNA and *Prox1* (Prospero homeobox protein 1) mRNA with real-time polymerase chain reaction (PCR), the extracted RNA was converted to cDNA by reverse transcription using the Tetro cDNA synthesis kit (Bioline). Levels of *Tie2* and *Prox1* transcripts were analysed using SensiFAST™ SYBR® kit (Bioline) with custom primers. *Prox1* primer was designed to amplify the proximal promoter region, including a forward primer of 5′-GCG CGC GGT ACC CCA GAT GTT TGC AAC ATA TA-3′ and a reverse primer of 5′-GCG CGC CTC GAG GCA GGA GAA AGA AGG AAA GG-3′. For *Tie2* PCR amplification, the primer sequence 5′-AGT TCG AGG AGA GGC AAT CA-3′ (sense) and 5′-CCG AGG TGA AGA GGT TTC CT-3′ (anti-sense) was selected. Evaluation of relative threshold cycle was performed by using endogenous human beta actin.

### Statistical Methods

The results were expressed as mean values ± standard deviations (mean ± SD). A two-way analysis of variance (ANOVA) was performed with post hoc testing (Tukeys' test) as appropriate to determine whether there were significant differences among the test conditions. A p-value < 0.05 was considered statistically significant.

## Results and Discussion

A miniature mouth gag and tongue suction system was developed to perform radiotherapy in the oral cavity of murine models while sequentially using micro-endoscopy at the same position non-invasively, as described in the “Methods” section. A schematic illustration of the setup for oral radiotherapy and intra-vital monitoring is shown in Figure 1(a). Wild-type and transgenic mice with green fluorescent protein (GFP) expressed specifically in blood and lymphatic vessels (*GFP-tie2* and *GFP-prox1*, respectively), were monitored before radiation was administered, and then in five day intervals afterward, as depicted in Figure 1(b). Changes in the blood and lymphatic vessels were visible at the cellular level using confocal endo-microscopes, as was the flow of individual red blood cells.

**Figure 2:**
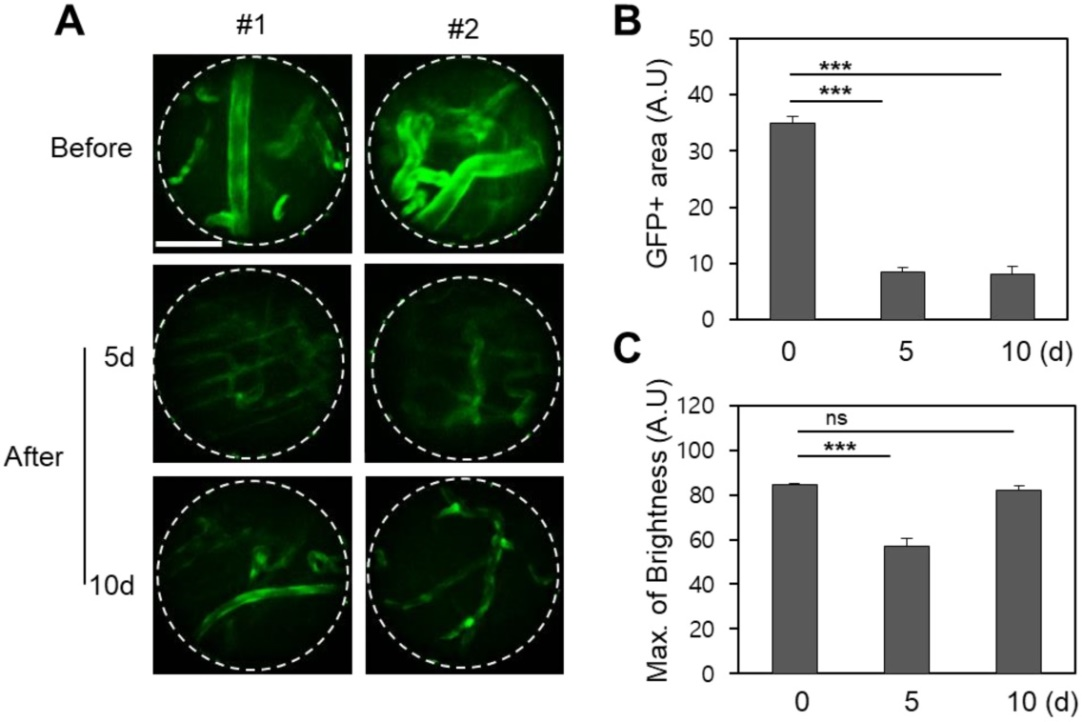
Changes in the blood vessels of the buccal mucosa after irradiation in *GFP-tie2*+ transgenic mice. (A) The GFP area and brightness were measured at the same site before irradiation and on days 5 and 10 after irradiation, as shown in representative endomicrographs. White lines outline the viewing area of the endoscope. (B) The area of green fluorescence ​​was observed to decrease significantly 5 and 10 days after irradiation. (C) The GFP intensity observed after irradiation decreased until day 5 and recovered by day 10. Scale bars, 100 µm. ns, non-significant; ***, p < 0.001.

The angiopoietin-1 receptor, also known as *tie2*, is a receptor protein with important roles in vascular development and angiogenesis. The *GFP-tie2+* mouse expresses green fluorescence at the blood vessel endothelium, enabling visualization which is highly useful for hemodynamic studies 24. *In vivo* endoscopic imaging of the mouse oral cavity was demonstrated for these mice using a 1.0 mm diameter GRIN micro-endoscope probe, and clearly reveals the physiological effect of a single fractionated 10 Gy radiotherapeutic dose on the oral vascular vessels in Figure 2. Compared to control images taken before radiation therapy, images taken 5 and 10 days after radiation from *GFP-tie2+* transgenic mice showed significantly decreased total green fluorescence area and maximum fluorescence brightness in the mean single endoscopic field of view (FOV), an observation which was attributable to a decrease in the vessel diameter (n = 5). A statistically significant decrease in area (p < 0.001) was observed from before radiation treatment to day 5 after treatment, and remained until day 10 after treatment. A concurrent decrease in maximum GFP fluorescence signal was also significant to p < 0.001 on day 5, and then rebounded to be indistinguishable from the original GFP intensity on day 10. Thus local recovery of fluorescence hints at damage and recovery of vascular endothelial cells. The fluorescence micrographs shown were selected from two mice to be representative of the observed changes in vascular tissue over the course of the treatment and follow-up.

**Figure 3:**
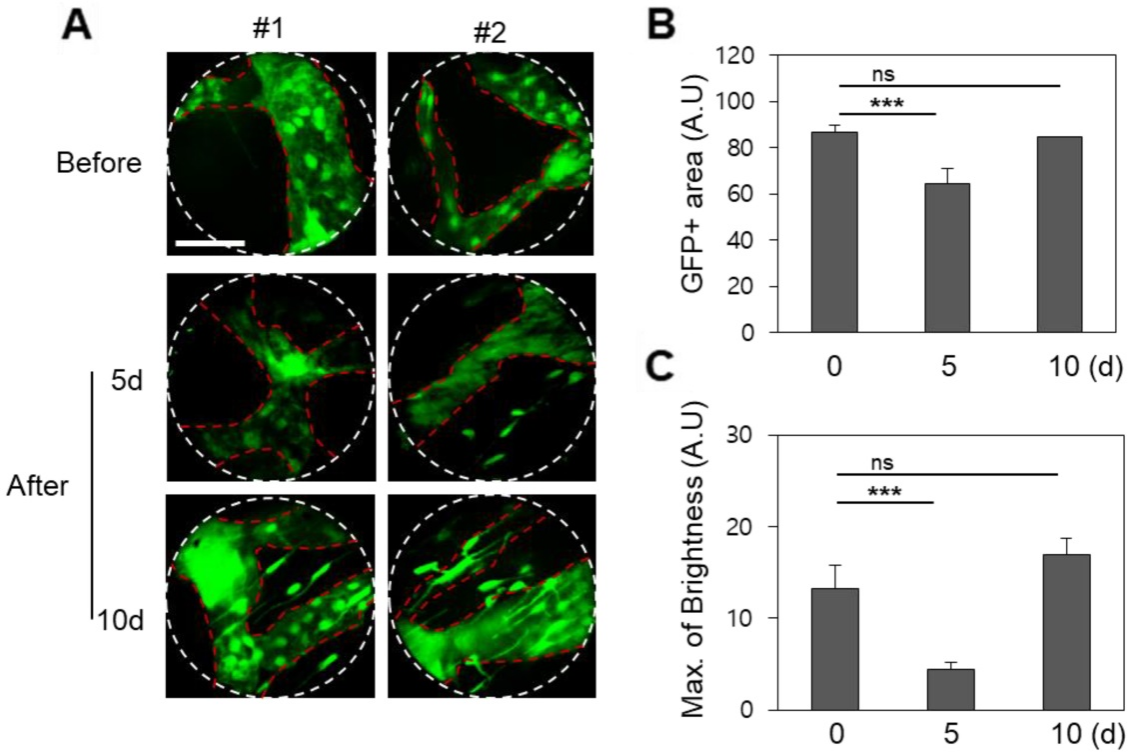
Changes in the lymphatic vessels after radiotherapy of the buccal mucosa of the *GFP-prox1* transgenic mice. (A) The GFP area and brightness were measured at the same site before irradiation and on days 5 and 10 thereafter, as shown in representative endomicrographs. Red dashed lines outline the lymphatic vessels, and white lines outline the viewing area of the endoscope. (B) The area of GFP observed after irradiation had decreased significantly by day 5 but recovered by day 10. (C) The ​​GFP intensity had significantly decreased 5 and 10 days after irradiation. Scale bars, 100 µm. ns, non-significant; ***, p < 0.001.

The *prox1* gene is a master control gene for lymphatic development, and transgenic mice expressing *GFP-prox1* express lymphatic-specific fluorescence. Using these mice, fluorescence confocal micro-endoscopic images of the lymphatic vessels were obtained by the same protocol, and were also analysed 5 and 10 days after treatment. Endoscopic micrographs are shown for two representative *GFP-prox1+* mice in Figure 3. Images of the lymphatic vessels similar to endoscopic angiography images were obtained, and the light source and PMT conditions were maintained across observations. Unlike in the vasculature, the observed total GFP area in the single endoscopic FOV decreased slightly to day 5 and then recovered again by day 10 (p < 0.001). This was a result of the active diameter of the lymphatic vessels decreasing between radiation treatment and day 5, and recovering to the pre-treatment conditions by day 10. The maximum value of the GFP signal was also modulated in a similar manner. The maximum observed fluorescence intensity decreased significantly on day 5 after radiation therapy, but had recovered by day 10, to be statistically indistinguishable from the original signal.

**Figure 4:**
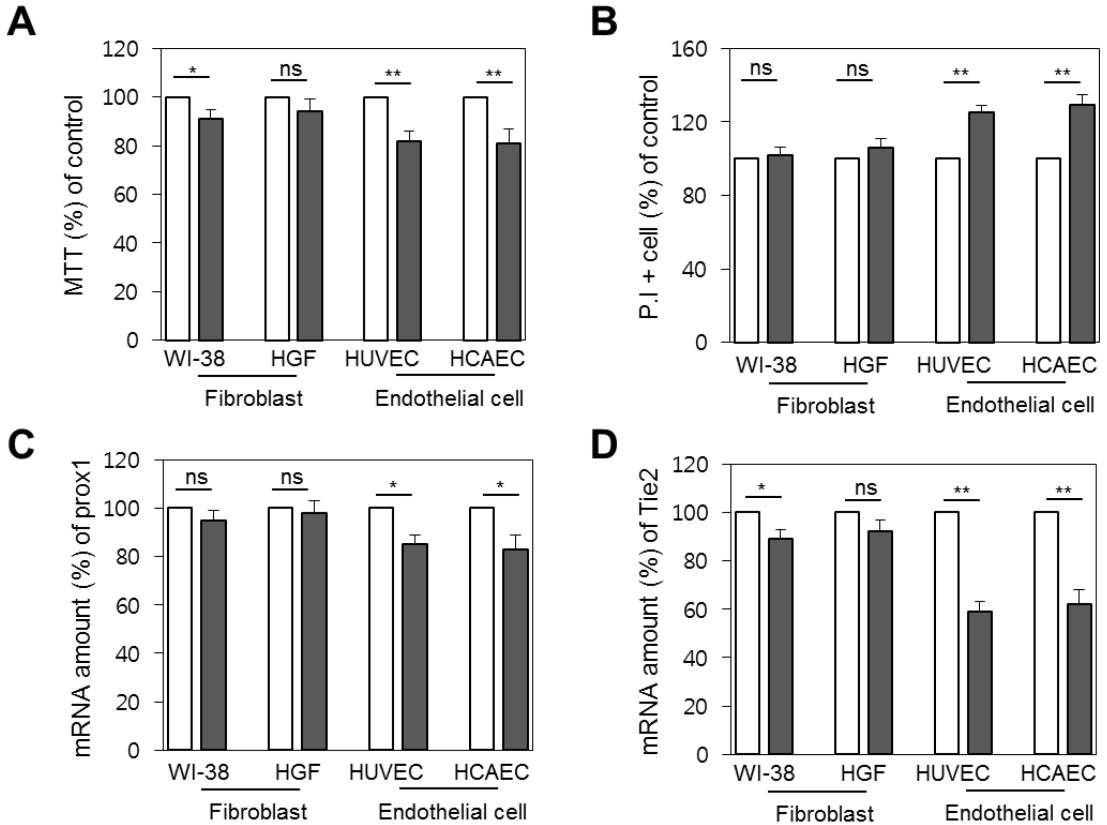
Vulnerability of endothelial cells upon radiation. Comparison of cellular viability between primary fibroblasts and endothelial cells before radiation treatment (white bars) and one day after (filled bars). (A) Cellular viability by MTT assay. (B) Cell death level by propidium iodide staining. (C) *Prox1* and (D) *tie2* gene expression as quantified by real time PCR. ns, non-significant; * , p < 0.05; **, p < 0.01.

To check whether the decrease in brightness was related to vascular and lymphatic endothelial cell death, we measured the difference in cellular viability between primary fibroblast, gingival fibroblast, and endothelial cells by 3-(4,5-dimethylthiazol-2-yl)-2,5-diphenyltetrazolium bromide (MTT) assay and propidium iodide (PI) staining. Although the cells commonly showed lower cell viability 1 day after radiation, the difference was only significant in endothelial cells, with an 18% drop in viability measured by MTT assay and 29% by PI staining (p < 0.01), as shown in Figure 4 (a) and (b), respectively. Further, as we thought that expression conditions of *tie2* or *prox1* might result in the reduction of imaging brightness, we verified the expression level of the genes after radiation exposure by real-time PCR. Interestingly, expression of the genes was reduced one day after radiation. Whereas the *prox1* gene expression pattern decreased to a significance to p < 0.05 in endothelial cells, at a similar rate to the cell death level (Figure 4c), the reduction of *tie2* expression was more prominent and significant at p < 0.01. (Figure 4d).

**Figure 5:**
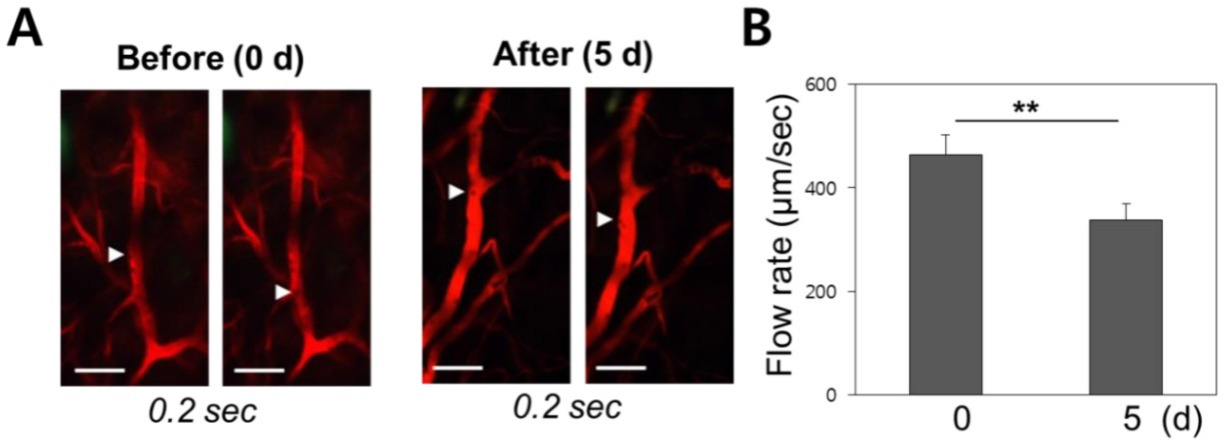
Change of mean blood flow velocity after irradiation. (A) Erythrocyte velocity within a blood vessel was measured using differential imaging over a 0.2 second time interval. (B) Red blood cells migrated at a speed of 450 µm/sec before radiation therapy. The flow speed decreased to 380 µm/sec after treatment. Scale bars, 100 µm; **, p < 0.01.

Vascular damage and repair should be correlated with blood flow velocity. Video from *in vivo* micro-endoscopy was used to analyse the hemodynamics of the blood flow before and after radiotherapy in wild-type mice. Rhodamine dextran was intravenously injected to allow visualization of the blood vessels, and the mean blood flow velocity of red blood cells was measured to have a mean of 450 ± 41µm/sec pre-treatment (n = 5), and to decrease down to 380 ± 32 µm/sec as observed on day 5 after radiation therapy (n = 5) (Figure 5). The observed decrease in vascular flow velocity following radiotherapy is significant at p < 0.01.

**Figure 6:**
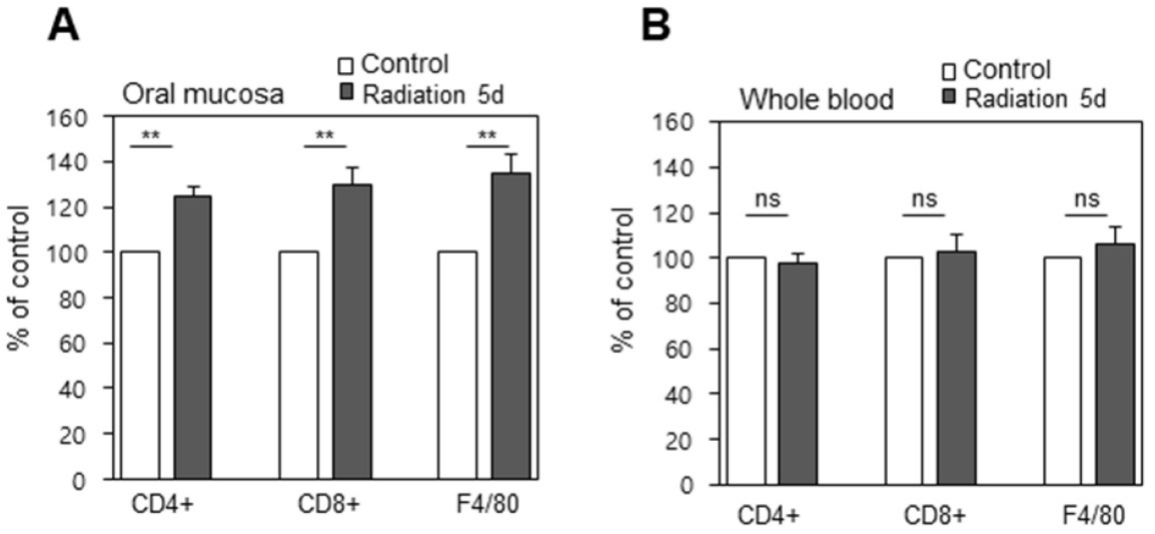
Infiltration of immune cells into the tissues (A) Immunofluorescence positive cells increased significantly after treatment (B) Blood from the same animals was isolated and the cells positive with the same markers were counted by FACS. ns, non-significant; **, p < 0.01.

Immunostaining revealed significant immune cells infiltration into the buccal mucosa, which was significant because it does not occur for the blood as a whole. Histological evaluations were performed to observe the immune response to radiation therapy. Immuno-fluorescence staining for CD4+, CD8+, and F4/80 cells were assessed from the buccal mucosa and from whole blood before and on day 5 after treatment, and total cell counts were assessed by fluorescence assisted cell sorting (FACS). This revealed the counts of CD4, CD8, and F4/80 from the oral cavity to be significantly (p < 0.01) increased, indicative of an immune response on day 5 after irradiation (Figure 6a), while whole blood samples didn't display a significant difference in cell lymphocyte counts before and after radiation (Figure 6b). This may be explained by the infiltration of immune cells due to tissue damage, deformation, and necrosis caused by irradiation, and this inflammation reaction may lead to complications if not controlled.

Overall, the combination of *in vitro* assays, RNA expression assays, and immune infiltration studies with fluorescence micro-endoscopy at the cellular level allows significant results to be drawn from the *in vivo* observations. While fibroblast cells and endothelial cells are quite hardy to radiotherapy *in vitro*, *in vivo* they express significant decreases in fluorescence intensity. This luminescence is more readily recovered in lymphatic vessels than in vascular tissues, in agreement with the observed difference of RNA expression in *prox1+* and *tie2+* cells following radiotherapy. Immunostaining shows significant infiltration of immune cells into the buccal mucosa which does not occur for the blood as a whole. The results suggest that the acute and long-term side effects of radiotherapy are amenable to longitudinal micro-endoscopic observation.

## Conclusion

Cranial radiation therapy is indispensable in the management of primary and metastatic brain tumours and head and neck cancer. However, brain irradiation is associated with several acute and late toxicity risks, which should be recognized and discussed during pre-treatment counselling sessions with patients for whom brain radiation is recommended. Radiotherapy complications are generally divided into acute side effects, early-delayed effects, and late effects. Acute side effects can occur during radiation treatment or at most 6 weeks after radiation, while early-delayed effects occur up to 6 months after radiation, and late effects can occur more than 6 months after completion of treatment 1,2. Unlike most reversible acute and initial delayed reactions, late reactions are generally not reversible. The acute effects of radiation are observed during the treatment process. Some of the more common side effects include temporary deterioration of basic neurological symptoms due to brain edema, fatigue, nausea and vomiting, dermatitis, and hair loss 5,6,25,26. Rare acute reactions include conductive mild myelosuppression, mucositis, and parotitis 7,8,27-30. Early-delayed responses that may occur months after brain radiation include transient focal neurologic symptoms (i.e., pseudoprogression) with increased or decreased MRI contrast enhancement. Inflammation and blood-brain barrier destruction can also indirectly cause cell damage.

In this study, we examined the effects of radiation therapy based on the changes of the blood or lymphatic vessels in the buccal mucosa of mice through a micro-endoscopic system. Based on our observations, the blood and lymphatic vessels and immune system were changed. In addition, anatomical deterioration was noted, and this anatomical injury caused functional problems after the irradiation. This may cause mucositis and parotitis, which are considered to be early complications. At the same time, these results can be used as the basis for delayed complications, such as cerebrovascular conditions, including occlusive vascular disease (ischemic stroke) and intracerebral cavernous malformations, which may cause intracranial bleeding.

Based on the results of the present study, the visualization of the vasculature of the buccal mucosa may be helpful in predicting clinical complications in patients after radiotherapy. The proposed technique can be used for early diagnosis and treatment of diseases in the future. Moreover, it is helpful in monitoring various physiological changes and understanding disease mechanisms.

## Abbreviations

CNS: central nervous system
FACS: fluorescence assisted cell sorting
FOV: field of view
GFP: green fluorescent protein
GRIN: gradient index
MTT: 3-(4,5-dimethylthiazol-2-yl)-2,5-diphenyltetrazolium bromide
PCR: polymerase chain reaction
PI: propidium iodide
PMT: photomultiplier tube
p*rox1*: Prospero homeobox protein 1
*tie2*: TEK receptor tyrosine kinase
